# Rethinking Long-Term Follow-Up of CPAP Therapy in Obstructive Sleep Apnea: Toward Personalized and Integrated Care

**DOI:** 10.3390/healthcare14152410

**Published:** 2026-08-05

**Authors:** Antonio Fabozzi, Francesca Romana Manfredi, Noemi Ascarelli, Laura Melis, Ilaria Domenica Piscopo, Federica Olmati, Ambra Nicolai, Arianna Sanna, Caterina Antonaglia, Alessia Steffanina, Matteo Bonini, Paolo Palange

**Affiliations:** 1 Pulmonology Unit, Department of Public Health and Infectious Diseases, Policlinico Umberto I, “Sapienza” University of Rome, 00185 Rome, Italy; francescaromana.manfredi@uniroma1.it (F.R.M.); noemi.ascarelli@uniroma1.it (N.A.); laura.melis@uniroma1.it (L.M.); ilaria.piscopo@uniroma1.it (I.D.P.); f.olmati@policlinicoumberto1.it (F.O.); a.nicolai@policlinicoumberto1.it (A.N.); a.sanna@policlinicoumberto1.it (A.S.); a.steffanina@policlinicoumberto1.it (A.S.); matteo.bonini@uniroma1.it (M.B.); paolo.palange@uniroma1.it (P.P.); 2 Pulmonology Unit, Department of Medical Surgical and Health Sciences, Hospital of Cattinara, University of Trieste, 34149 Trieste, Italy; caterinaantonaglia@gmail.com

**Keywords:** obstructive sleep apnea, adherence, compliance, continuous positive airway pressure, follow-up, long-term care

## Abstract

**Background**: Although the diagnostic and treatment pathway for Obstructive Sleep Apnea (OSA) is well established, long-term management is still not standardized. **Methods**: We conducted a narrative review of studies published between 2000 and 2026 in PubMed/MEDLINE. **Results**: Continuous Positive Airway Pressure (CPAP) adherence and healthcare resource allocation remain the real critical issue of long-term management for OSA patients. Early proactive interventions like phone calls and early medical visits may be particularly valuable for improving CPAP nightly usage. Telemedicine, educated and trained home-care providers or primary care can lead to improved adherence, long-term monitoring and earlier identification of treatment-related issues. Patient-reported outcomes, comorbidities, endotypes, and phenotypic stratification should be integrated into follow-up management. **Conclusions**: OSA long-term care should shift from a device-centered approach toward a personalized, patient-centered model with the integration of multidimensional risk stratification, multidisciplinary care, telemedicine and primary care support.

## 1. Introduction

Obstructive sleep apnea (OSA) is a chronic and multifactorial respiratory disease with high prevalence characterized by recurrent upper airway collapse during sleep, resulting in intermittent hypoxia and sleep fragmentation [1]. This disease affects approximately 936 million people worldwide, representing a serious public health problem [2]. OSA pathophysiology involves complex neurobiological and genetic underpinnings that extend beyond simple mechanical airway obstruction [3]. Dysregulation of neurotransmitter systems plays a pivotal role, with patients often exhibiting elevated serum dopamine levels that may function as a compensatory mechanism to regulate arousal, respiratory drive, and upper airway muscle tone in response to intermittent hypoxia [4]. Genetic variations further modulate disease expression; specifically, the T allele of the DRD2 rs1800497 polymorphism has been identified as an independent predictor of higher arousal rates and increased OSA severity [4]. Additionally, OSA is linked to the disruption of circadian clock genes, such as the down-regulation of neuronal PAS domain protein 2 (NPAS2), which interacts with hypoxia-inducible factors and is associated with the metabolic dysregulation observed in these patients [5]. OSA causes a spectrum of clinical consequences, including excessive daytime sleepiness (EDS), cognitive impairment, and mood disorders [6,7]. It also leads to an increased risk of morbidity and mortality and significant healthcare costs, related to its strong association with cardiovascular and metabolic comorbidities, such as hypertension, heart failure, atrial fibrillation and diabetes [8,9]. Although several international and national guidelines provide recommendations for the follow-up of patients with OSA, the optimal timing, intensity, content, and organization of long-term care remain incompletely standardized and vary across healthcare systems [10,11]. In particular, evidence supporting personalized, risk-stratified follow-up pathways for patients receiving Continuous Positive Airway Pressure (CPAP) remains limited.

In clinical practice, follow-up is guided by data derived from CPAP use, particularly the residual Apnea–Hypopnea Index (AHI), parameters indicative of adherence to therapy and clinical questionnaires such as Epworth Sleepiness Scale (ESS) [12,13]. However, these data do not include the broader clinical context. Indeed, since OSA is a heterogeneous pathological condition, relying exclusively on CPAP-derived parameters could overlook important features such as symptoms, comorbidities and individual patient characteristics and could limit the ability to personalize treatment. 

At the same time, new digital technologies such as telemedicine, telemonitoring and artificial intelligence are rapidly growing in the OSA landscape. These tools enable remote data collection, early identification of treatment-related issues and patient adherence difficulties [14]. These tools thus appear to improve treatment adherence and optimize healthcare resources [15]. However, they have not yet been incorporated into actual clinical practice protocols.

In light of these considerations, this narrative review aims to provide an updated overview of long-term management strategies and follow-up approaches in patients with OSA treated with CPAP therapy. Particular attention will be given to emerging models of care based on personalized, multidimensional and multidisciplinary frameworks, with the goal of moving beyond a purely device-centered approach toward a more comprehensive and personalized management paradigm.

## 2. Materials and Methods

A structured literature search was conducted using PubMed/MEDLINE, Scopus, Web of Science and the Cochrane Library to identify articles published between 1 January 2000 and 1 May 2026. The search strategy included terms associated with OSA long-term follow-up, adherence to PAP therapy, healthcare organization, digital health, patient-reported outcomes and personalized management. The main search terms included ‘obstructive sleep apnoea’ or ‘obstructive sleep apnoea’ and ‘continuous positive airway pressure’ or ‘CPAP’ or ‘positive airway pressure’ or ‘PAP’ in combination with ‘follow-up’, ‘long-term management’, ‘long-term care’, ‘adherence to continuous positive airway pressure’, ‘treatment persistence’, ‘telemedicine’, ‘telemonitoring’, ‘remote patient monitoring’, ‘primary care’, ‘home care’, ‘integrated care’, ‘patient-reported outcomes’, ‘phenotype’, ‘endotype’, ‘arousal threshold’ or ‘loop gain’. The search was focused on peer-reviewed articles published in English and involving adult populations. To identify further relevant publications, the reference lists of eligible studies, systematic reviews, clinical guidelines, consensus documents and major narrative reviews were manually examined. Articles were suitable if they involved adults with OSA and addressed at least one of the following domains: early or long-term follow-up after the start of PAP treatment; adherence, persistence or discontinuation of PAP therapy; residual symptoms; telemedicine or remote PAP monitoring; primary care, home care, multidisciplinary or ‘hub-and-spoke’ care models; patient-reported outcomes or experiences; clinical, phenotypic or endotypic stratification; or personalized treatment and transition to alternative therapies. Clinical guidelines, consensus statements, systematic reviews, meta-analyses, randomized controlled trials and prospective or retrospective observational studies were considered. Preprints and other non-peer-reviewed sources were considered only when they addressed emerging areas for which peer-reviewed evidence was limited. These sources were explicitly identified as preliminary and were not used as the sole basis for clinical recommendations.

Duplicate records were removed prior to selection. Titles and abstracts were assessed independently by two reviewers, followed by an assessment of the full text of potentially eligible articles. For each included study, we collected details about the study design, the population, the sample size, the intervention or care model, the duration of follow-up, the outcomes assessed, and the main findings relevant to the long-term management of OSA. The methodological relevance of each study was assessed in relation to the specific claims made in this narrative review. We paid special attention to whether the study evaluated an actual follow-up intervention or merely reported an association; whether it included an appropriate comparison group; whether it evaluated adherence, symptoms, quality of life, clinical outcomes or healthcare resource use; whether sufficient consideration had been given to confounding factors; and whether the proposed strategy was feasible and generalizable to routine clinical practice. Greater importance was assigned to guidelines, systematic reviews, meta-analyses, randomized controlled trials and large-scale prospective studies. Data from observational studies were used to address areas where no interventional evidence was available, whilst emerging findings in the fields of physiology and digital health were interpreted as generating hypotheses where their clinical implementation had not been prospectively validated. As part of the quality control strategy, studies were assessed for eligibility by more than one author; numerical results were cross-checked against the full-text original publications, and the strength of each claim was determined according to study design, consistency, the strength of the evidence and clinical feasibility. No formal meta-analysis was carried out, nor was any assessment of risk of bias at study level applied, due to the narrative design and the heterogeneity of the literature. The manuscript was critically prepared according to the domains of the Scale for the Assessment of Narrative Review Articles (SANRA).

## 3. Results

### 3.1. Why Follow-Up Is as Important as Therapy in OSA

OSA, in addition to being a widespread pathological condition, presents multiple short- and long-term consequences that contribute to the development and progression of multiple morbidities. Short-term consequences of OSA include a worsening of the metabolic profile, primarily with weight gain, and systemic arterial hypertension, as well as excessive daytime sleepiness and a poor quality of life [16,17,18].

Long-term consequences of OSA, particularly when untreated or inadequately treated, include a significantly increased risk of cardiovascular and cerebrovascular diseases. Marin et al. demonstrated that untreated severe OSA was associated with an odds ratio (OR) of 2.87 for fatal cardiovascular events and 3.17 for non-fatal cardiovascular events compared with healthy controls [19]. Yaggi et al. further reported that OSA was independently associated with a hazard ratio (HR) of 1.97 for stroke or death after adjustment for confounding variables [20]. More recent meta-analytic evidence confirmed a severity-dependent relationship between OSA and cardiovascular risk, with pooled HRs progressively increasing from 1.21 in mild OSA to 2.45 in severe disease [21]. This cardiovascular risk is not static but dynamically evolves over time according to disease severity, cumulative nocturnal hypoxic burden, associated comorbidities, and, most importantly, long-term treatment adherence [22].

Persistent sleep fragmentation and intermittent hypoxia adversely affect attention, executive function, psychomotor vigilance, and cardiovascular regulation, thereby contributing to both individual morbidity and increased healthcare burden [13]. The importance of follow-up in OSA arises from the fact that CPAP, the gold-standard treatment for moderate-to-severe OSA, has consistently demonstrated efficacy in reducing EDS, improving sleep quality and health-related quality of life, and lowering blood pressure values [23]. Importantly, several studies suggest that the cardiovascular protective effect of CPAP is strongly dependent on adequate long-term adherence, particularly ≥4 h/night of use [21]. Clinical trials have shown that only CPAP-adherent patients exhibited significant reductions in systolic and diastolic blood pressure [24]. Furthermore, a recent post-hoc multi-trial analysis demonstrated that CPAP reduced the risk of major adverse cardiovascular and cerebrovascular events (MACCE) by 35% in the high-risk OSA population (i.e., those with a high hypoxic burden or high delta heart rate) [25]. Long-term adherence also affects healthcare costs for OSA patients. Large-scale retrospective data show that adherent patients have significantly fewer emergency room visits (0.39 vs. 0.54 for non-adherent patients) and fewer all-cause hospitalizations (0.09 vs. 0.13) in the first year of therapy [26]. Crucially, maintaining adherence is a matter of public safety; CPAP therapy has been shown to dramatically reduce daytime sleepiness, lowering ESS scores from an average of 12.6 to 3.6, which effectively eliminates traffic accidents and improves five quality-of-life dimensions, including physical and social functioning, vitality, and general health perception [27,28].

However, CPAP adherence in real-world settings remains suboptimal, particularly during the first months after treatment initiation. Average long-term adherence in routine clinical practice is frequently reported around 4–5 h/night, while long-term discontinuation rates may exceed 30–40% [29,30]. Poor adherence is associated with persistent residual symptoms, ongoing excessive daytime sleepiness, increased cardiovascular risk, and higher healthcare utilization [22]. In this context, structured follow-up plays a pivotal role in achieving meaningful long-term outcomes.

### 3.2. Factors Influencing Follow-Up

One of the major factors affecting the follow-up of OSA is CPAP adherence. Early identification of treatment-related side effects, including mask intolerance, air leaks, nasal or oral dryness, pressure discomfort, and nocturnal awakenings, may be particularly valuable, particularly during the first weeks of treatment, when long-term adherence trajectories are often established [31].

Regarding differences in terms of CPAP adherence between different populations, the effect of age is still debated. Some authors have shown that older individuals are more likely to use CPAP, whereas others have reported lower adherence to therapy [32]. In a study by Palm A et al., each 10-year increase in age was associated with a 13% lower relative risk of CPAP non-adherence versus full adherence (adjusted relative risk ratio [aRRR] 0.87), after adjustment for sex, BMI, AHI, ESS, hypertension, and humidifier use [33]. OSA severity also impacts CPAP adherence: the risk of CPAP discontinuation drops by 20% for every 10-unit increase in AHI (aRRR 0.80) [33]. EDS is another factor influencing CPAP adherence: the risk of CPAP withdrawal decreases by 4% per every unit increase in ESS (aRRR 0.80) [33]. Obesity also has a significant impact on CPAP adherence. Indeed, it appears that non-obese OSA patients have lower adherence, and some authors have suggested that this is due to the stronger association between non-obese OSA and the low arousal threshold endotype, which is known to be linked to poor treatment adherence [34]. A Swedish registry-based cohort study demonstrated that an increasing BMI up to 35 was associated with higher CPAP adherence [33]. Regarding socioeconomic factors, a stable relationship, a high income and a high educational level are associated with higher nightly CPAP usage (additional 13.2–20.5 min of nightly use) [35].

Sex- and gender-related differences also influence follow-up. Women with OSA are more likely to present with residual symptom burden after treatment initiation, due to stronger association with anxiety, depression, and comorbid insomnia [36]. A nationwide Danish cohort study has shown that women have higher morbidity and mortality rates than men, as well as higher social welfare costs both before and after a diagnosis of OSA [37]. Furthermore, the evidence regarding CPAP adherence between men and women is highly debated: while some studies show higher adherence among women, others demonstrate the opposite [38,39]. These differences are likely due to variations in the methods used to select the study population. Adherence to CPAP is also influenced by ethnicity; in fact, the average nightly usage rate among the Black population is approximately 1 to 1.5 h lower than that of the White population [40]. Overall, one of the current challenges in OSA care is the need to move from a uniform, CPAP-centered follow-up model toward a more individualized and phenotype-driven approach, integrating adherence monitoring, residual disease burden, comorbidities, sex/gender differences, and patient-centered outcomes (Table 1).

### 3.3. The Impact of Follow-Up Timing

**Figure 1:**
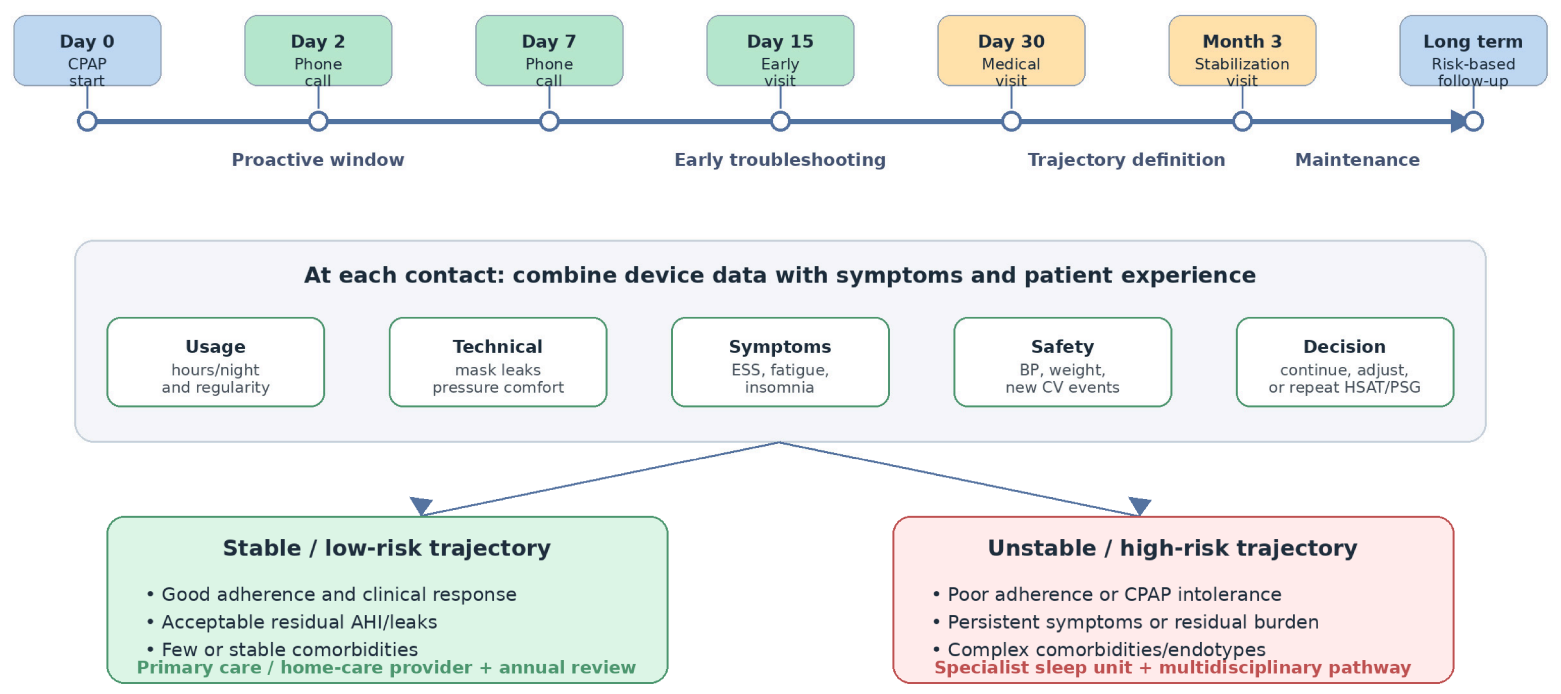
Critical first 90 days of CPAP follow-up in Obstructive Sleep Apnea. The figure illustrates a pragmatic follow-up pathway from CPAP initiation to three-month reassessment, including early proactive contact, clinical troubleshooting, multidimensional evaluation, and subsequent allocation to standard or intensified long-term follow-up. Abbreviations: AHI, apnea–hypopnea index; BP, blood pressure; CPAP, continuous positive airway pressure; CV, cardiovascular; ESS, Epworth Sleepiness Scale; HSAT, home sleep apnea test; PSG, polysomnography.

The first week of CPAP therapy appears to be the strongest predictor of long-term treatment adherence. Data from the literature indicate that an initial follow-up visit at 1 month is too late to prevent CPAP discontinuation [41]. Indeed, 98% of patients who are non-adherent at three months have already this pattern by day 30 [42]. Conversely, the study by Bouloukaki et al. demonstrated that an early intervention strategy involving proactive phone calls at days 2 and 7 and follow-up visits on days 15 and 30 significantly improves both nightly usage (6.9 vs. 5.2 h) and treatment regularity (92.8% vs. 79.8%) over 24 months [43]. Furthermore, a single home visit around day 16 can boost nightly usage by 60 to 102 min for patients with low initial adherence [44]. Therefore, considering this evidence and in order to reduce long-term cardiovascular risks, the first follow-up visit should ideally occur within weeks 1 to 3 (Figure 1).

### 3.4. Instrumental Follow-Up

The instrumental follow-up for OSA remains a controversial topic, and guidelines have not yet fully addressed this issue. The residual AHI calculated by PAP devices is based on the identification of reductions in airflow, whereas when using PSG during PAP therapy, other equally important parameters are taken into account, such as sleep efficiency, the percentage of deep sleep, arousals, lower limb movements and oxygen desaturations [45]. In this regard, the study by Fanfulla F et al. highlighted precisely this issue: 28.4% of OSA patients classified as ‘well-controlled’ using the AHI derived from the PAP device were ‘not well-controlled’ when the AHI calculated via PSG was used [46]. The authors hypothesised that the main reason underlying this significant difference is the poor effectiveness of PAP devices in identifying hypopneas during therapy (sensitivity for detecting hypopneas ranging from 58% to 74% and falling even further in cases of high pressure) [47].

Furthermore, there is another limitation regarding OSA follow up using data from PAP devices: 6.6% of patients experienced a change in residual AHI of more than 5 events per hour when switching from one brand of PAP device to another [48]. Considering all this evidence, the latest 2021 AASM guidelines recommend a PSG or HSAT during PAP therapy for patients who remain symptomatic despite good adherence to treatment, for those with a change in body weight of at least 10%, or for those who develop new clinical conditions, particularly cardiovascular ones [49].

### 3.5. Endotypes and Adherence

Emerging evidence suggests that OSA endotypes may contribute to CPAP adherence. A low respiratory arousal threshold (ArTH) has been associated with lower treatment adherence: in patients with coronary artery disease (CAD), a 1-standard deviation (SD) reduction in ArTH was associated with a 49-min decline in nightly use [50]. Similarly, a recent non-peer-reviewed preprint reported that the ‘Low ArTH Driven’ endotype, identified by Latent Profile Analysis (LPA), has been associated with significantly lower treatment adherence during the first three months of follow-up [51]. 

The high loop gain (LG) endotype has been associated with a threefold higher risk of having a residual AHI ≥ 10 events/hour at follow-up, although longer nightly CPAP usage [52]. Potentially this evidence may be due to the larger rebound in REM sleep experienced by these patients when treated. The high upper airway collapsibility endotype has been associated with greater hourly CPAP usage [53].

These findings suggest that physiological traits may provide additional information regarding early treatment tolerance and residual respiratory instability. However, the available evidence remains predominantly observational, and it has not yet been demonstrated that assigning patients to different follow-up pathways according to endotypic characteristics improves adherence or clinical outcomes. Furthermore, physiological endotyping requires specific signals, additional costs, analytical methods and software that are not routinely available in all sleep centres. Endotypic information should therefore currently be considered an adjunct to conventional clinical assessment rather than a prerequisite for determining follow-up intensity (Table 2).

### 3.6. The Impact of Telemedicine in CPAP Adherence

The role of telemedicine (TM) in the follow-up of OSA patients is complex, varied and controversial, yet at the same time promising. A recent meta-analysis of randomized controlled trials suggests that TM significantly improves adherence to CPAP, with an increase in nightly usage between 29.25 and 34.2 min [54]. Fridriksson et al. demonstrated significantly higher adherence with proactive telemedicine follow-up compared with conventional management (4.3 ± 2.4 vs. 4.1 ± 2.6 h/night), together with significantly lower mask leakage rates (5.4% vs. 12.1%) [55]. TM-based education programmes alone are insufficient to improve CPAP adherence, whereas the Tele-OSA trial demonstrated that sending automated feedback messages improved adherence at 90 days (4.4–4.8 h vs. 3.7 h with usual care) [56]. Other clinical trials, such as TELEPAP and OPTISAS1, have shown that TM is more effective in difficult-to-treat patients or those at high cardiovascular risk, whereas it appears to have little effect in easy-to-treat patients and those at low cardiovascular risk [57,58]. TM also makes it possible to proactively identify any emerging issues during the first few months of treatment. TM monitors residual AHI, mask leaks and PAP levels with high accuracy [13,59]. The AlertApnèe study has shown that telemonitoring can identify Cheyne–Stokes respiration, which is known to be associated with heart failure [60]. A non-peer-reviewed white paper issued by a healthcare consulting firm estimated that telemedicine-based care pathways may reduce healthcare costs by approximately USD 2743 per patient per year; however, this estimate should be interpreted cautiously because it has not been independently validated in a peer-reviewed economic evaluation [61]. A 12-month RCT confirmed that delegating follow-up to a Remote Medical Centre can successfully increase nightly use in patients with initial adaptation difficulties (mean nightly CPAP use increased from 2.5 ± 0.8 to 4.3 ± 1.6 h/night) while completely eliminating the need for in-person visits to the specialized OSA Unit, thereby significantly reducing specialist workload [59]. In a study by Riney et al., a TM-based digitalized OSA care pathway enabled an initial consultation with a sleep specialist within 5 days from screening; after a further 12 days, HSAT was performed; after a further 9 days, the patient received an OSA diagnosis with treatment recommendations; and after a further 8 days, CPAP therapy was started (meaning approximately one month elapsed from screening to the start of therapy) [62]. Despite all these potential benefits, telemedicine-based follow-up is still a long way from becoming standard practice in many geographical areas, due to healthcare organisation and technological infrastructure. The current limitations to the implementation of telemedicine in the long-term management of OSA include the need for trained personnel, integration with electronic health records, and clear protocols to avoid omissions or duplication of workflow. The varying use of telemedicine across different parts of the world is also due to differences in reimbursement policies. Indeed, in some countries, telemonitoring activities, remote clinical review, home-care provider involvement, and digital education are not uniformly recognized or reimbursed across healthcare systems. Obviously, this limits its use. Finally, patients’ digital and technological literacy should also be considered as one of the limitations of telemedicine in the follow-up of OSA. Limited familiarity with technology, lack of access to the internet or compatible devices, cognitive decline, language barriers and privacy issues represent further limitations to the implementation of telemedicine (Table 3).

## 4. Discussion

**Figure 2:**
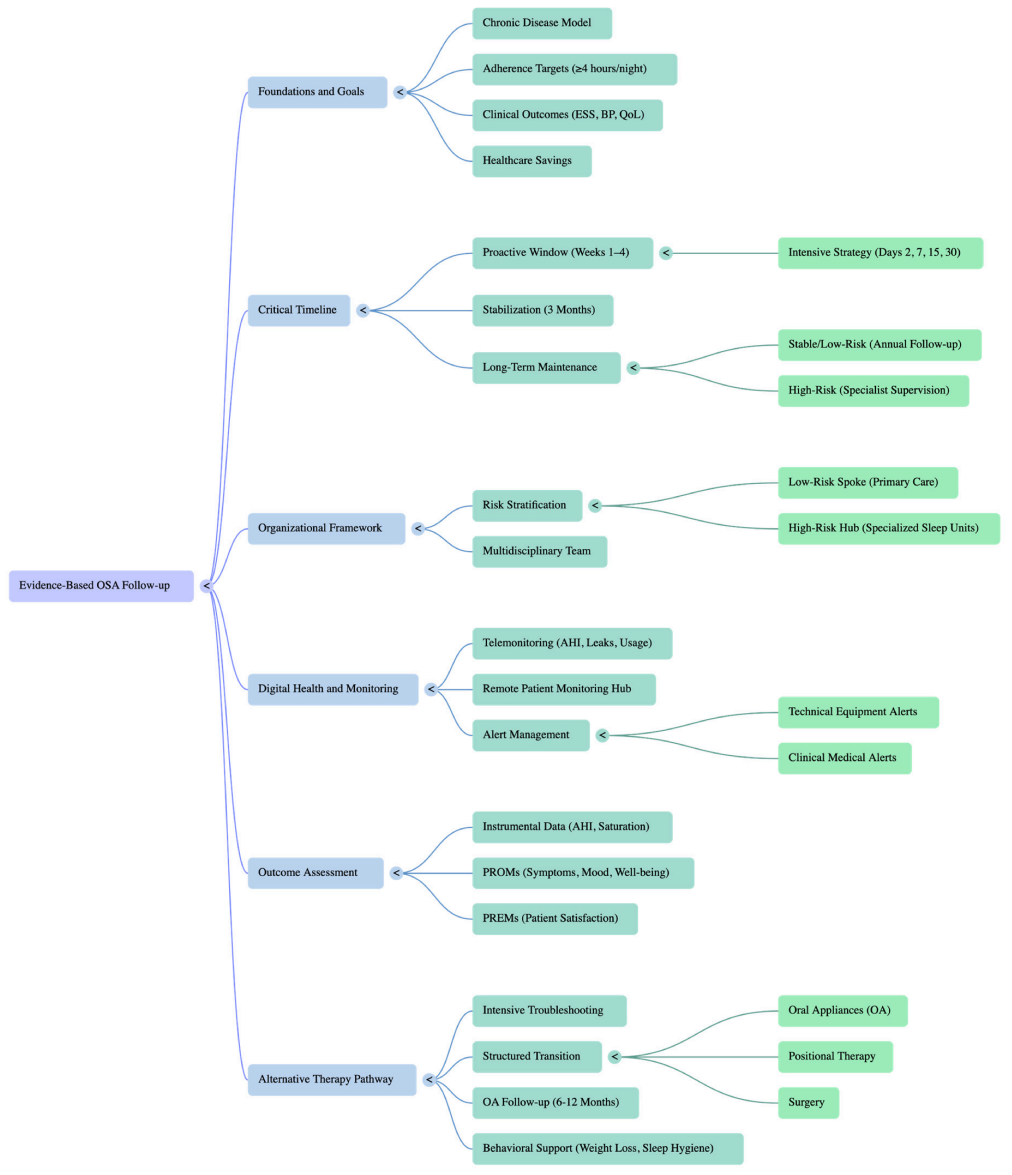
Proposed evidence-based follow-up in Obstructive Sleep Apnea. The figure summarizes a structured, multidimensional model for long-term management of patients with obstructive sleep apnea (OSA). The framework integrates the goals of follow-up; the critical timeline; the organizational model based on risk; digital health tools, including telemonitoring, remote monitoring hubs, and alert management; patient-reported outcome measures (PROMs); patient-reported experience measures (PREMs); and alternative therapy pathways. Abbreviations: AHI, apnea–hypopnea index; BP, blood pressure; ESS, Epworth Sleepiness Scale; OA, oral appliance; OSA, obstructive sleep apnea; PREMs, patient-reported experience measures; PROMs, patient-reported outcome measures; QoL, quality of life.

In light of all the evidence that has emerged from our narrative review, we propose a structured follow-up framework comprising six domains: long-term care goals, the follow-up timeline, multidimensional assessment, the organisation of care based on low- or high-risk patients, telemedicine, and personalised patient care (Figure 2). The follow-up we propose should not be interpreted as a rigid protocol to be applied in every context, but should rather be tailored to local financial resources, the availability of experienced specialists and the organisation of the local healthcare system. The first principle of our framework concerns the objectives of our structured follow-up. First and foremost, OSA must be regarded as a chronic condition and managed using a model that takes this into account. The first real issue to be addressed in follow-up should be the adherence to CPAP therapy, as poor adherence is the primary risk factor for residual symptoms, cardiovascular events, a poor quality of life and a waste of healthcare resources [29,30]. For this reason, it is essential to verify that CPAP is used correctly for at least 4 h per night, every night. Furthermore, the effectiveness of therapy should not be assessed solely on the basis of data such as residual AHI, but also by evaluating residual sleepiness, blood pressure and quality of life. The ultimate aim is to establish a chronic care model that improves adherence to therapy, eliminates symptoms and reduces healthcare costs. The second element of our framework concerns the timing of follow-up.

Scheduling the follow-up appointment directly after one month of therapy is too late to prevent patients from quitting CPAP therapy [41,42]. We propose a proactive intervention strategy, involving two phone calls in the first week and a medical visit in the second week. This approach seems to increase nightly CPAP usage and long-term treatment adherence [43]. The aim of these calls should be to early address some issues such as mask intolerance, excessive leaks, nasal or oral symptoms, and pressure discomfort. This early phase should be followed by a stabilization assessment at approximately three months, when the patient’s trajectory can be more reliably defined. Finally, for the long-term management, we propose a ‘hub-and-spoke’ model (third component of our model), which has also been proposed by other authors [31]. Indeed, patients with uncomplicated OSA, who demonstrate high treatment adherence and have few comorbidities, can be managed effectively within primary care with an annual medical visit (plus eventually the support of home care providers), as studies have shown comparable outcome rates to those of specialist centres in terms of symptom control and treatment adherence [63]. Conversely, highly specialised multidisciplinary units (hubs) should prioritise complex cases, such as patients with unstable cardiovascular disease, residual excessive daytime sleepiness, Overlap Syndrome, high-risk endotypes such as high loop gain and low arousal threshold, central sleep apnea, stroke or concomitant insomnia (COMISA), which require multidisciplinary supervision and targeted interventions such as cognitive behavioural therapy for insomnia (CBT-I), non-invasive ventilation or adaptive-and-servo-ventilation (ASV) [31,63]. The fourth component of our proposed organisational framework concerns the use of digital care and telemedicine in the long-term management of patients. Remote access to CPAP usage, leaks, pressure levels, and residual AHI can facilitate the early identification of technical problems and support timely troubleshooting. In this context, the future lies with the ‘Remote Patient Monitoring (RPM) Hub’, which also involves properly trained and educated home-care providers [63]. This model integrates data downloaded remotely from the CPAP device with physiological variables measured by wearables and smartphones, such as blood pressure, physical activity, sleep patterns and heart rate variability [63]. The fifth step in the follow-up protocol we propose involves assessing the effectiveness of CPAP therapy. The residual AHI is certainly a very important factor to assess, but as we have seen previously, it can misclassify as ‘well-controlled’ in around 30% of patients [46]. 

For this reason, we propose including Patient-Reported Outcome Measures (PROMs) and Patient-Reported Experience Measures (PREMs) [63]. It is necessary to monitor daytime sleepiness using the Epworth Sleepiness Scale, as well as quality of life and patient satisfaction, in order to early identify patients who remain symptomatic despite adequate adherence to treatment. In this context, it may therefore be necessary to conduct secondary diagnostic investigations to identify depression, comorbid insomnia, endotypes such as a low arousal threshold, other non-respiratory sleep disorders or cardiorespiratory comorbidities. Finally, the framework should include a multidisciplinary structured pathway for patients who remain intolerant of CPAP or experience an inadequate clinical response despite optimization. Initial management may include mask reassessment, pressure adjustment, humidification, management of nasal symptoms, behavioural support, weight management, and treatment of concomitant insomnia. When these measures are insufficient, transition to oral appliance therapy, positional therapy, surgery, or alternative ventilatory modalities may be considered according to the underlying phenotype, comorbidities, and patient preferences. Such transitions should be planned and monitored rather than regarded as treatment failure or loss to follow-up.

Finally, it is important to emphasise that the proposed framework should be interpreted as an evidence-based conceptual model rather than as a prospectively validated clinical algorithm. The individual components of the framework are supported by varying levels of evidence. The early resolution of PAP-related issues and active telemonitoring have been evaluated in interventional studies, whilst the concept of the hub-and-spoke model, the multimodal remote monitoring and the remote patient monitoring hub are supported mainly by observational studies or expert consensus. Furthermore, there is currently no consensus about how patients should undergo standard rather than intensified follow-up. The proposed stratification should therefore remain pragmatic and be based primarily on routinely available information, including early use of CPAP, treatment tolerance, residual symptoms, comorbidities and patient-reported outcomes. Prospective studies should assess whether the integrated approach improves treatment adherence, clinical outcomes, healthcare utilisation and cost-effectiveness compared with standard care.

### Knowledge Gaps and Future Research Priorities

Several knowledge gaps have so far limited the development of standardised long-term follow-up protocols. Firstly, the optimal timing, frequency and intensity of follow-up visits are still poorly defined. Although early proactive interventions appear to be beneficial, pragmatic comparative studies are needed to identify which patients would benefit most and which interventions—telephone contact, telemonitoring, home visits or early specialist evaluation—are most effective and sustainable.

Secondly, there are currently no universally accepted criteria to identify which patients should be assigned to standard rather than intensive follow-up. Future studies should develop and externally validate pragmatic stratification tools based on routinely available variables, including early use of PAP, treatment tolerance, residual symptoms, comorbidities, weight change and patient-reported outcomes. The added value of physiological endotyping, and measurements obtained via wearable devices and artificial intelligence should therefore be assessed beyond this clinically accessible baseline assessment. Thirdly, future clinical trials should go beyond the device-derived residual AHI to include symptoms, quality of life, treatment experience, cardiovascular and metabolic outcomes, use of healthcare resources, treatment withdrawal, and transition to alternative therapies. Finally, the clinical efficacy, cost-effectiveness and equity of telemedicine and ‘hub-and-spoke’ models should be assessed prospectively across different healthcare systems. Implementation studies should take into account infrastructure, reimbursement, staffing requirements, alert burden, digital literacy and inequalities in access. Older people, women, socio-economically disadvantaged populations and patients with multimorbidity should be adequately represented to ensure that personalised follow-up does not inadvertently exacerbate existing inequalities.

## 5. Conclusions

OSA should be considered a chronic and heterogeneous condition requiring long-term, patient-centered management rather than a single diagnostic and therapeutic intervention. Although CPAP remains the first-line treatment, poor adherence and treatment discontinuation remain major barriers, particularly during the first weeks of therapy. Therefore, structured and proactive follow-up, including early patient contact, timely troubleshooting and collaboration with home-care providers, is essential. Telemedicine and remote patient monitoring may further support personalized care by integrating device data with symptoms, comorbidities and patient-reported outcomes. However, residual AHI derived from PAP devices should not be considered sufficient to define treatment success, as it may underestimate persistent respiratory events and overlook clinically relevant symptoms. A hub-and-spoke model may help reserve specialist sleep centres for complex phenotypes while allowing stable patients to be managed in primary care. Future studies should validate standardized, phenotype-driven follow-up pathways to improve adherence, outcomes and sustainability of care.

## Figures and Tables

**Table 1:** Factors influencing CPAP adherence and follow-up in Obstructive Sleep Apnea.

Factor	Literature Evidence	Evidence Type and Consistency	Follow-Up Implications
Age	The effect of age remains debated; however, registry data reported a 13% lower risk of CPAP withdrawal for every 10-year increase in age (aRRR 0.87) [32,33].	Registry-based observational evidence; findings across studies are inconsistent.	Age should not be used alone to define follow-up intensity; it should be integrated with symptoms, comorbidities and early usage patterns.
OSA severity	The risk of CPAP discontinuation decreases by approximately 20% for every 10-event/h increase in AHI (aRRR 0.80) [33].	Registry-based observational evidence; association reported in a large cohort but not prospectively validated as a risk-stratification criterion.	Patients with mild or minimally symptomatic OSA may require stronger education and motivational support.
Excessive daytime sleepiness	Higher ESS scores are associated with a lower risk of CPAP withdrawal, with an estimated 4% reduction per ESS point [33].	Observational evidence; association with persistence does not establish a causal effect.	Residual sleepiness should be monitored because persistent symptoms despite adequate use may require reassessment.
BMI and obesity phenotype	Increasing BMI up to 35 kg/m^2^ was associated with higher CPAP adherence, whereas non-obese OSA may show poorer adherence [33,34].	Observational evidence; findings may vary across populations and OSA phenotypes.	Non-obese phenotypes may need closer early follow-up and phenotype-driven interventions.
Socioeconomic status	Stable relationship, higher income and higher educational level were associated with an additional 13.2–20.5 min of nightly CPAP use [35].	Observational evidence; susceptible to residual confounding and healthcare-system effects.	Follow-up pathways should include education, accessibility support and simplified patient–device communication.
Sex and gender	Women more often present residual symptom burden related to anxiety, depression and comorbid insomnia; evidence on sex-related adherence differences remains conflicting [36,38,39].	Observational evidence; findings regarding PAP adherence are conflicting.	PROMs, insomnia screening and mood assessment should be systematically included in follow-up.
Ethnicity	Average nightly CPAP use among Black patients has been reported to be approximately 1–1.5 h lower than among White patients [40].	Observational, context-dependent evidence, mainly derived from US cohorts.	Equity-oriented models should address access barriers, health literacy and tailored support.

AHI, apnea–hypopnea index; aRRR, adjusted relative risk ratio; BMI, body mass index; CPAP, continuous positive airway pressure; ESS, Epworth Sleepiness Scale; OSA, obstructive sleep apnea; PAP, positive airway pressure; PROMs, patient-reported outcome measures; US, United States.

**Table 2:** OSA endotypes and their relationship with CPAP adherence and residual burden.

Endotype	Literature Evidence	Evidence Type and Consistency	Potential Follow-Up Implications
Low respiratory arousal threshold (low ArTH)	(1) A 1-SD reduction in ArTH was associated with a 49-min decline in nightly CPAP use in patients with coronary artery disease [50]. (2) The Low ArTH Driven endotype was characterized by significantly lower CPAP adherence during the first three months of follow-up [51].	Observational evidence; the Low ArTH Driven profile was reported in a non-peer-reviewed preprint.	When available, low ArTH identification may suggest closer follow up; consider comorbid insomnia or anxiety. Not validated for routine clinical follow-up.
High loop gain	High loop gain was associated with a threefold higher risk of residual AHI ≥10 events/h during follow-up, but with longer nightly CPAP use in some cohorts [52].	Observational and prognostic evidence; no prospective evaluation of loop-gain-guided follow-up.	Do not rely on adherence alone; monitor residual AHI, breathing instability and persistent symptoms, and consider further instrumental evaluation when needed. Not validated for routine clinical follow-up.
High upper airway collapsibility	High upper airway collapsibility was associated with greater hourly CPAP usage [53].	Observational evidence; findings require confirmation in prospective and external cohorts.	May be associated with greater perceived benefit from CPAP. However, current evidence is insufficient to define a specific follow-up pathway on the basis of collapsibility alone.

AHI, apnea–hypopnea index; ArTH, arousal threshold; CPAP, continuous positive airway pressure; OSA, obstructive sleep apnea; SD, standard deviation.

**Table 3:** Telemedicine evidence and clinical implications for long-term follow-up in Obstructive Sleep Apnea.

Telemedicine Domain	Literature Evidence	Evidence Type and Maturity	Potential Clinical Implications	Limitations
Overall effect on CPAP adherence	Increase in nightly CPAP use of approximately 29.25–34.2 min [54].	Meta-analytic evidence from randomized trials, supported by individual RCTs; effect size is generally modest and heterogeneous.	Telemedicine may support adherence, particularly when embedded into structured follow-up rather than used as isolated education.	The magnitude of benefit is moderate and depends on intervention design, patient selection and baseline adherence risk.
Early proactive telemedicine follow-up	Increased nightly use from 4.1 ± 2.6 to 4.3 ± 2.4 h/night and decrease in mask leakage from 12.1% to 5.4% [55].	Interventional evidence; benefit depends on the design and intensity of the accompanying clinical support.	Remote early contact may identify technical problems before they become reasons for discontinuation.	Clinical value may be greater when combined with rapid troubleshooting, mask optimization and patient-tailored feedback.
Automated feedback and patient messaging	CPAP use increased to 4.4–4.8 h/night compared with 3.7 h/night with usual care [56].	Device-validation and observational evidence; clinical outcome benefit from alert-based management remains less established.	Behavioral reinforcement and feedback loops appear more effective than passive education alone.	Digital education should not replace active monitoring, feedback and clinical escalation pathways.
Remote device monitoring and alert management	Telemonitoring can accurately track residual AHI, mask leaks and PAP levels; AlertApnée showed that telemonitoring can identify Cheyne–Stokes respiration associated with serious cardiac events [13,59,60].	Randomized evidence in selected patients with poor initial adaptation; broader generalizability requires confirmation.	Remote monitoring can support technical alerts, clinical alerts and early reassessment when residual disease or new cardiorespiratory patterns emerge.	Industry-wide standardization of metrics remains limited; device-derived residual AHI should not be interpreted as a complete marker of control.
Remote Patient Monitoring/Remote Medical Centre model	A 12-month randomized controlled trial showed that delegating follow-up to a Remote Medical Centre improved mean nightly CPAP use from 2.5 ± 0.8 to 4.3 ± 1.6 h/night [59].	Randomized interventional evidence from a single study in a selected population; broader effectiveness and generalizability require confirmation.	Remote hubs may reduce specialist workload while maintaining proactive management of patients with early poor adaptation.	Requires trained home-care providers, predefined escalation rules and integration with specialist sleep units.
Economic sustainability	Estimated reduction of $2743 per patient per year [61].	Preliminary non-peer-reviewed economic estimate; not independently validated through a formal peer-reviewed cost-effectiveness analysis.	Telemedicine may improve sustainability of long-term OSA care when integrated into hub-and-spoke or RPM-based models.	Economic impact is likely to vary across healthcare systems. Infrastructure costs, reimbursement, workforce requirements, digital literacy, and unequal access may limit scalability and widen disparities.

AHI, apnea–hypopnea index; CPAP, continuous positive airway pressure; HSAT, home sleep apnea testing; OSA, obstructive sleep apnea; PAP, positive airway pressure; RPM, remote patient monitoring.

## Data Availability

No new data were created or analyzed in this study. Data sharing is not applicable to this article.
